# Testing hypotheses of developmental constraints on mammalian brain partition evolution, using marsupials

**DOI:** 10.1038/s41598-017-02726-9

**Published:** 2017-06-26

**Authors:** Alison Carlisle, Lynne Selwood, Lyn A. Hinds, Norman Saunders, Mark Habgood, Karine Mardon, Vera Weisbecker

**Affiliations:** 1 The University of Queensland, School of Biological Sciences, St. Lucia, 4072 QLD Australia; 2 The University of Melbourne, School of BioSciences, Parkville, 3010 VIC, Australia; 3 CSIRO Health and Biosecurity Flagship, Canberra, 2601 ACT, Australia; 4 The University of Melbourne, Pharmacology and Therapeutics, Parkville, 3010 VIC, Australia; 5 The University of Queensland, Centre of Advanced Imaging, St. Lucia, 4072 QLD, Australia

## Abstract

There is considerable debate about whether the partition volumes of the mammalian brain (e.g. cerebrum, cerebellum) evolve according to functional selection, or whether developmental constraints of conserved neurogenetic scheduling cause predictable partition scaling with brain size. Here we provide the first investigation of developmental constraints on partition volume growth, derived from contrast-enhanced micro-computed tomography of hydrogel-stabilized brains from three marsupial species. ANCOVAs of partition *vs*. brain volume scaling, as well as growth curve comparisons, do not support several hypotheses consistent with developmental constraints: brain partition growth significantly differs between species, or between developing *vs*. adult marsupials. Partition growth appears independent of adult brain volume, with no discernable growth spurts/lags relatable to internal structural change. Rather, adult proportion differences appear to arise through growth rate/duration heterochrony. Substantial phylogenetic signal in adult brain partitions scaling with brain volume also counters expectations of development-mediated partition scaling conservatism. However, the scaling of olfactory bulb growth is markedly irregular, consistent with suggestions that it is less constrained. The very regular partition growth curves suggest intraspecific developmental rigidity. We speculate that a rigid, possibly neuromer-model-like early molecular program might be responsible both for regular growth curves within species and impressions of a link between neurogenesis and partition evolution.

## Introduction

The structure and regionalization of the mammalian brain has its origins at the dawn of vertebrate evolution^1^. Superimposed on this ancient pattern was a dramatic evolutionary expansion during several events of somatosensory evolution^2^, which coincided with the evolution of a very large, inside-out patterned, six-layered neocortex. Understanding the patterning of brain size and regionalization has been directed at all levels of organization﻿, from gross brain size and proportions (macromorphology) to cellular structure. While these are often pursued separately^3^, increasing attention has been given to the integration of development, cell structure, and macroevolution, in a bid to arrive at a more unified theory of how mammalian brains could have evolved^4–7^.

**Figure 1:**
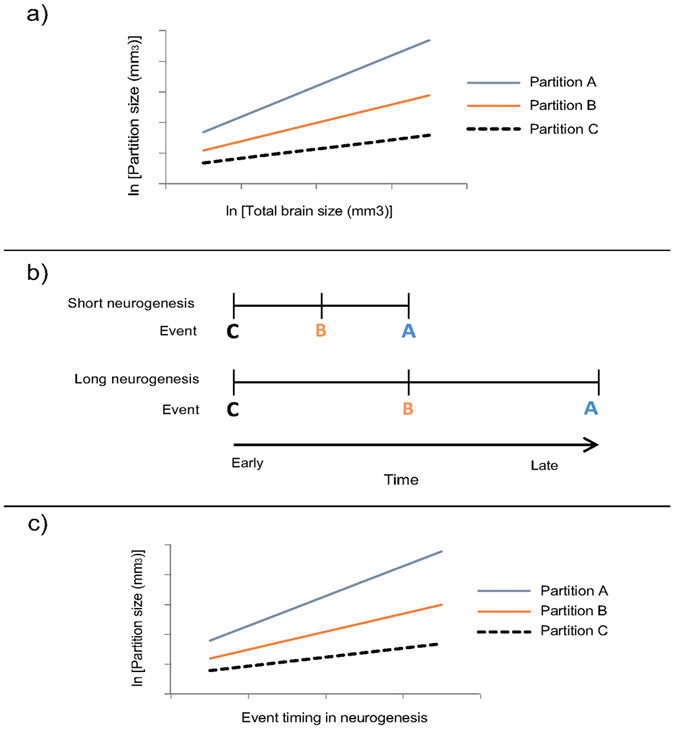
Schematic of the connection between partition scaling and neurogenesis in the evolution of brain proportions suggested by “late equals large”. Evolutionary partition scaling with brain size differs between A, B, and C (**a**), with steeper-scaling A and B also having longer and later-onset neurogenesis (**b**). “Late equals large” proposes that the neurogenetic timing directly predicts partition scaling (**c**).

A controversial but widely-cited hypothesis that integrates several aspects of brain evolution is known by its shorthand name “late equals large”^8^. It argues that developmental constraints cause the brain to evolve as a tightly integrated unit. “Late equals large” suggests a tight correlation of brain partition sizes with total brain size and each other (Fig. 1a). It also notes that neurogenesis (the formation of neurons from neuronal precursor cells) occurs later and lasts longer in larger brain partitions as well as in larger brains (Fig. 1b). Because extended neurogenesis produces more neural precursors, which divide multiple times to result in a larger adult neural cell population, “late equals large” suggests that larger brain partitions (e.g. the mammalian neocortex) arise through a mechanistic link of later and longer-lasting neurogenesis (hence “late equals large”) (Fig. 1c). This phenomenon has been suggested to occur across vertebrates^9, 10^.

For mammals, an important implication of “late equals large” is that brain proportions should not vary much according to other factors (e.g. adaptation to a particular sensory, locomotor or cognitive function) or covariation between brain components after accounting for variation in brain size. In particular, the dominant size of the neocortex in large-brained mammals would be a by-product or “spandrel”^11^, of the developmental processes that lead to the evolution of an overall larger brain, rather than a result of selective pressures for particular functions or behaviours^5, 8^, or vice versa (selection for a large neocortex might force the remainder of the brain to keep pace). However, later elaborations of “late equals large” have highlighted the potential for developmental timing differences (heterochrony) in neurogenetic events as a potential source for brain proportion divergence within mammals^7, 12^.

While the “late equals large” hypothesis is widely cited and supported^4, 10, 13^, it has not gone unchallenged^6, 7, 13–16^. In particular, the opposing hypothesis that brain partitions evolve in a mosaic fashion (where parts of the brain can evolve independently) has had substantial support from work on primates^17–19^, carnivorans^20^, across rodents^21^ and in mice only^6^, cetaceans^22^ as well other vertebrates^23, 24^, which show substantial volumetric independence of brain parts. In addition, the methods used to derive suggestions of conserved neurogenetic schedules^25^ have been criticized^14^. Investigations into the cellular patterning of early brain development^24, 26^, and new neurogenetic data^25, 27, 28^ have also resulted in a wealth of new cellular-level developmental data. These reveal associations between neurogenetic timing and brain partition size^12, 25^, but also show interspecific developmental variability which was at least initially not predicted by “late equals large”^12, 29^ and is more consistent with mosaic evolution of brain development^14^. There is also ample evidence that brain partition volume and cell content do not scale linearly, so that larger brains have fewer cells per volume^30^. The relationship between cell density and partition volume, and the way in which neurogenesis relates to brain proportion growth, is therefore at least more complicated than suggested by the initial outline of “late equals large” (but see Charvet *et al*.^28^ for criticisms of brain cell counts). Work on brain development in the gray short-tailed opossum *Monodelphis domestica* ^31^ also shows that neuron and cell density of several brain regions peak at very small brain sizes within the first postnatal month, which is inconsistent with direct impacts of neuronal or cellular density on partition size.

Due to the difficulties of linking cellular and macromorphological brain development, a mechanism by which a late/long neurogenesis might produce larger brain partitions remains unclear. However, “late equals large” implies that brain growth should be determined by patterns of neurogenesis in some form, so that an investigation of regularities in mammalian brain partition development represents a useful yet unexplored test of whether neurogenesis determines patterns of brain growth^7^. Several hypotheses would support developmentally-conserved and neurogenesis-driven brain scaling, which this study aims to investigate:

*Hypothesis 1: Conserved partition scaling between species mirrors evolutionary scaling*.

**Figure 2:**
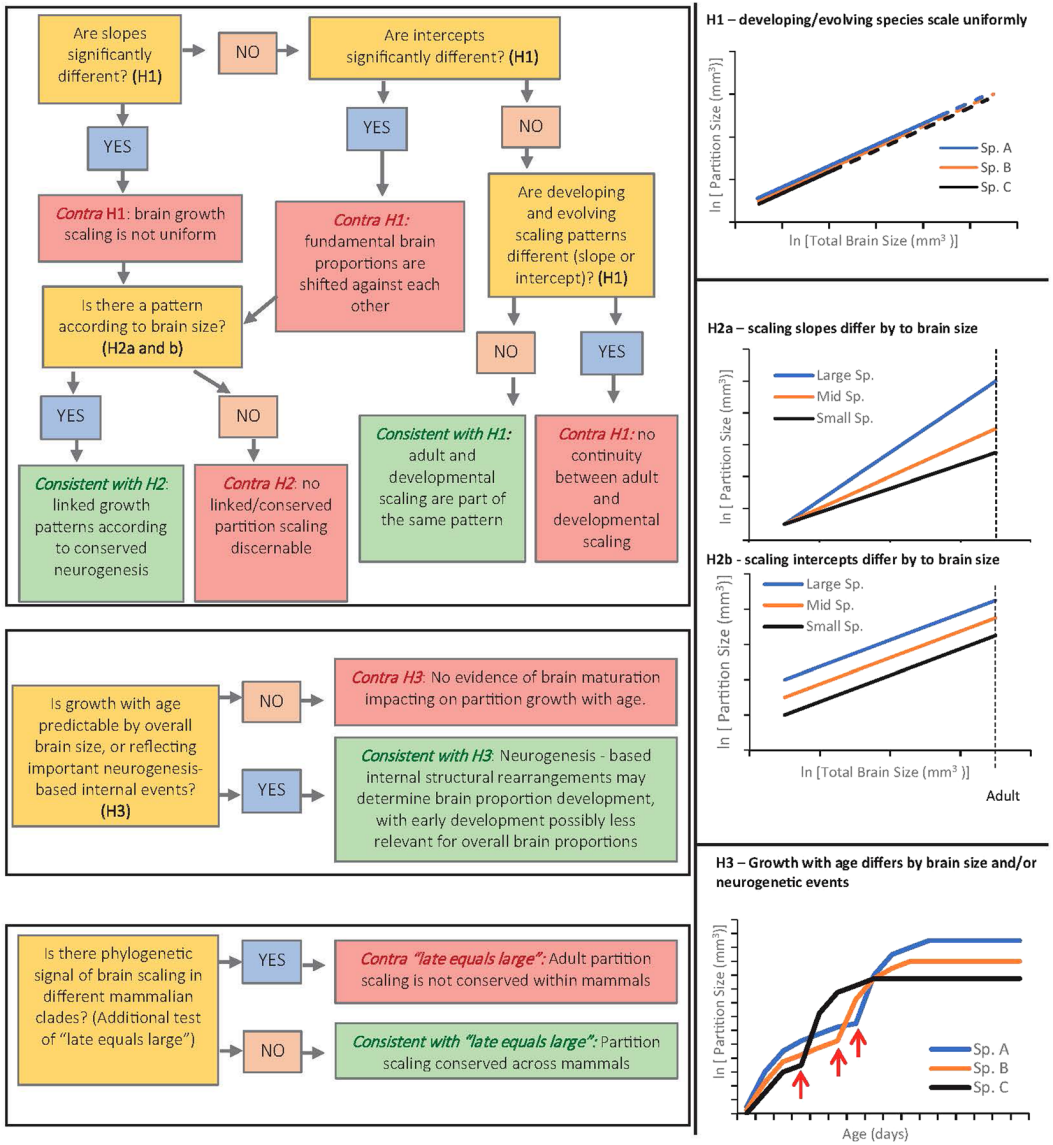
Flowchart outlining the hypotheses examined and interpretation of analyses conducted in this study (see introduction). H1, H2 and H3 refer to the three hypotheses for which accompanying graphs provide supporting visual representations: H1) Brain scaling slopes and intercepts are not significantly different between developing species, between evolving species (adult), or between developing and evolving brains (dotted lines signify continuation of brain scaling through adult brain sizes); H2a) and (**b**) example scenarios of a change in slope (H2a) or intercept (H2b) in the scaling of a partition according to brain size; and H3) all species growth patterns show growth irregularities attributable to neurogenesis and predictable by adult size (red arrows mark the occurrence of an important neurogenetic event as well as the onset of a growth spurt).

In the simplest case (Hypothesis 1 – H1), developmental partitions scale as a simple continuation of evolutionary partition scaling (see also Montgomery *et al*.^7^). Interspecific brain proportion differences would then be determined by brain proportions at termination of growth. H1 would provide the strongest support for “late equals large” because it reflects the suggestion that a predictable stretching of neurogenetic events causes predictable brain scaling. H1 predicts no significant difference between developmental and evolutionary partition scaling with brain size (Fig. 2), with larger partitions scaling more steeply than smaller ones^7^.

*Hypothesis 2*: *Non-uniform interspecific partition scaling, predictable by adult brain size*.

A slightly more complicated hypothesis (H2) predicts that developmental scaling might differ from evolutionary scaling, but that neurogenesis-directed partition growth results a linked, conserved scaling pattern that is predictable by adult brain size (Fig. 2). For example, larger-brained species might have higher intercepts or steeper slopes for the relatively larger partitions (e.g. cerebrum) compared to the relatively smaller ones (e.g. medulla).

*Hypothesis 3: Intraspecific growth patterns with age predictable by overall brain size, or growth reflecting important internal events*.

Conserved neurogenetic schedules might result in specific brain growth schedules with age, converging on a particular pattern according to brain size. For example, growth might parallel the conserved neurogenetic schedule hypothesized by “late equals large”, such that larger partitions start to grow later and for longer. In addition, brain regionalization occurs long before neurogenesis begins, and the neuronal-lineage cell populations of the brain show substantial growth only when the brain has already reached considerable size^27, 31^. “Late equals large” might therefore also be supported if there were two discrete stages in partition growth, an earlier one governed by different mechanisms and a second growth stage after neurogenesis has produced substantial cell numbers. Events that are related to neurogenetic completion or internal structural re-arrangements (e.g. at times when the brain receives new sensory inputs such as eye opening or leaving the pouch) might also result in growth spurts at particular times (Fig. 2).

### Phylogenetic signal in brain partition evolution as an additional test for conserved scaling

In addition to the developmental hypotheses outlined here, conserved developmental patterning has been suggested to result in uniform and linked pattern partition scaling across mammals, such that phylogenetic (ancestry-dependent) signal is only expected in unusual cases of neurogenetic heterochrony (e.g. differences in neurogenetic timing between primates and other mammals^29^). This has recently been qualified to include the possibility of grade shifts in neurogenesis and partition scaling between some mammalian clades^12, 27^, but cross-species comparisons of partition scaling have not explicitly accounted for phylogeny in the past^14, 16, 32^. The phylogenetic signal in brain proportion scaling can provide an important additional test of whether brain partition evolution is linked and conserved, or proceeds in a mosaic process^15, 33^.

Previous debates on whether brain proportions evolve under a developmental constraint have been based on repeated analyses of two data sources - adult partition/brain volume data and neurogenetic sequence data. Here, we add a new type of data to test the three developmental scenarios outlined above, using the first comparative dataset on mammalian brain partition growth sourced through a modified^34^ new method of diffusible iodine contrast-enhanced soft-tissue CT scanning (“DiceCT”)^35^. We use marsupial mammals due to the ease of accessibility of early brain growth and their uniform developmental patterning^36^, which precludes much of the developmental diversity and growth variation in placental clades^37^. We combine these data with published information on adult marsupial brain proportions to compare the allometry of within-species scaling and between-species adult proportions, and to test for phylogenetic signal in brain partition evolution. This allows us to provide a comparative view of partition development, using a straightforward ANCOVA workflow, which for the first time permits the integration of partition growth and partition evolution patterns.

## Results

### Regression plots of partition volume against age and size

**Figure 3:**
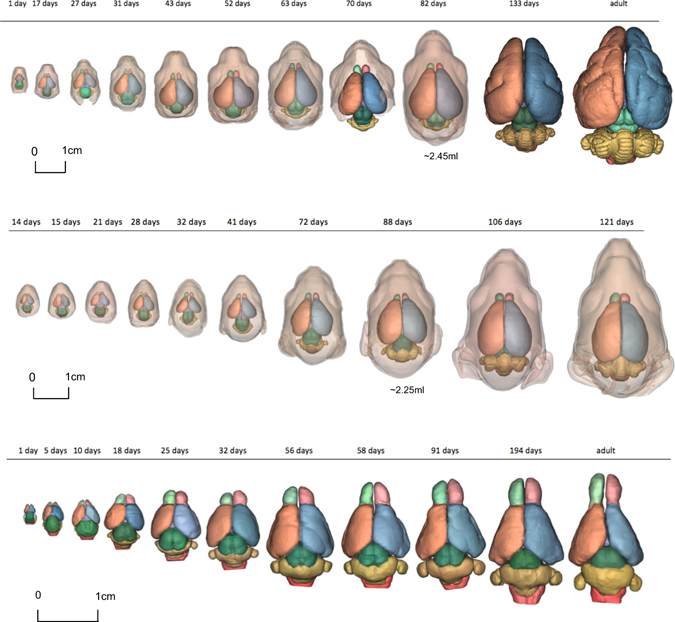
Chart of 3D-reconstructed dissected brains and, where available, head outlines used in this study. Green/light red, the two olfactory bulbs; orange/blue, cerebral hemispheres; dark green, midbrain; yellow, cerebellum; cherry red, medulla.

**Figure 4:**
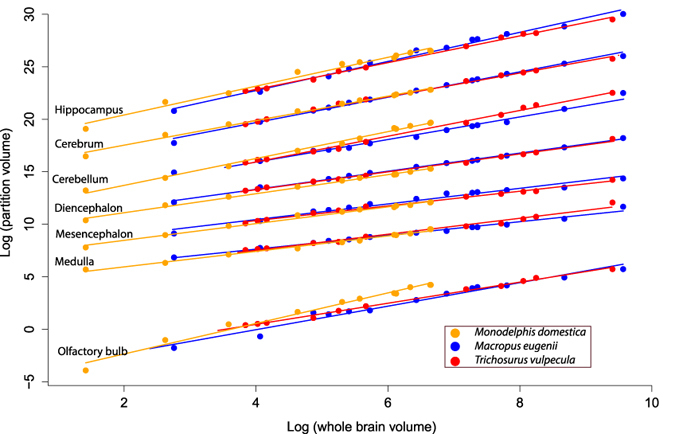
Joint plot of log partition volume against log brain volume (in mm^3^), with linear regression lines. Artificial intercepts (arbitrarily raising the slopes so they are not superimposed on the image) were added to allow separate viewing of the regression lines as established by^8^. The regression lines in this plot were cropped in Adobe illustrator.

Developmental series of 3D reconstructed *M. eugenii*, *T. vulpecula* and *M. domestica* brains are displayed in Fig. 3; the 3D data are accessible on MorphoSource (morphosource.org; Project ID 300) in stl format, and the volume data from the reconstructions are available in Supplementary Data File S1. Linear fits of brain partition size vs. whole brain volume are shown in Fig. 4, with separate graphs also available in Supplementary Fig. S2. For the scaling exponents for the three species, the joint developmental dataset and the dataset for adult marsupials, see Table 1.

**Table 1:** Intercepts, slopes, R^2^ and significance values of linear regressions of log partition sizes vs.

Partition	All developing			Macropus eugenii			Trichosurus vulpecula			Monodelphis domestica			Adults (marsupials)		
	**Int**.	**Sl**.	**R** ^**2**^	**Int**.	**Sl**.	**R** ^**2**^	**Int**.	**Sl**.	**R** ^**2**^	**Int**.	**Sl**.	**R** ^**2**^	**Int**.	**Sl**.	**R** ^**2**^
Olf. Bulb	−3.99	1.1	0.92	−4.5	1.12	0.97	−3.41	1	0.99	−5.27	1.47	0.98	−0.16	0.66	0.72
Cerebr.	−1.81	1.29	0.98	−2.14	1.4	0.98	−1.54	1.24	0.97	−1.59	1.25	0.99	−1.84	1.2	0.98
Hippoc.	−5.31	1.32	0.98	−5.85	1.4	0.99	−5.27	1.28	0.99	−5.34	1.38	0.98	−1.5	0.89	0.97
Dienc.	−1.23	0.88	0.99	−0.87	0.84	0.996	−0.86	0.83	1	−1.5	0.89	0.99	−2.38	1	0.99
Midbrain	−0.57	0.74	0.97	−0.28	0.72	0.97	−0.22	0.67	0.99	−0.9	0.77	0.99	−0.69	0.75	0.97
Cerebell.	−3.45	1.15	0.97	−3.34	1.08	0.98	−4.92	1.25	0.99	−3.86	1.31	0.99	−1.78	1	0.99
Medull.	0	0.68	0.98	0.37	0.61	0.98	−0.17	0.73	0.98	−0.08	0.67	0.98	−1	0.86	0.99

whole brain volume minus partition sizes. All regressions are significant at *p* < 0.0001.

### Scenarios 1,2 – uniform growth and scaling in development and evolution or other possibly neurogenesis-related growth regularities

ANCOVAs of partition volume and whole-brain volume minus partition size (Table 2) revealed diverse interspecific scaling patterns across partitions (for the near-identical results of partition volume against whole-brain volume, see Supplementary Table S2). The only brain partitions with no significant slope or intercept differences between species were the cerebral hemispheres and possibly the medulla; for the medulla, the interaction term of slope and species was close to the significance cut-off (0.03) with no significant pairwise differences in intercepts between species, so we tentatively accept that it might not differ substantially between species and conform to the predictions of H1. Subsequent analysis of developmental and adult scaling of cerebra and medulla revealed significant differences in intercepts (Table 2); note however that we cannot exclude the possibility that this intercept difference is due to systematic differences in sampling method between adult and developing datasets. Among the remaining partitions, olfactory bulbs and cerebellum growth scaling differed in slopes between our three species. Counter to H2, the smallest-brained *M. domestica* had a cerebellar scaling slope intermediate between *M. eugenii* and *T. vulpecula*. Of the remaining partitions, growth scaling intercepts differed in the diencephalon, hippocampus, and midbrain. The two relatively closely related *M. eugenii* and *T. vulpecula* tended to differ less from each other than either did from *M. domestica*, although they were substantially different in cerebellar growth slope and significantly different in midbrain intercepts.

**Table 2:** ANCOVA results for partition volume growth patterns against whole brain minus partition volume.

	ANCOVA	Olfactory bulb	Cerebrum	Hippocampus	Diencephalon	Midbrain	Cerebellum	Medulla
	*All 3 species*	*p* < 0.0001***	*p* = 0.066	*p* = 0.149	*p* = 0.083.	*p* = 0.1134	*p* = 0.001**	*p* = 0.03*
	*M. eug. – M. dom*.	*p* < 0.0001***	No sig. pairwise differences				*p* < 0.0001***	*p* = 0.28
Slope	*M. dom. – T. vulp*.	*p* < 0.0001***					*p* = 0.000***	*p* = 0.037*
	*T. vulp. – M. eug*.	*p* = 0.0356*					*p* = 0.003**	*p* = 0.23
Intercept	*All 3 species*		*p* = 0.783	*p* = 0.001**	*p* < 0.0001***	*p* = 0.001**		0.067.
	*M. eug. – M. dom*.			*p* = 0.029*	*p* < 0.0001***	*p* = 0.001**		No sig. pairwise differences
	*M. dom. – T. vulp*.			*p* = 0.001**	*p* < 0.0001***	*p* = 0.32		
	*T. vulp. – M. eug*.			*p* = 0.363	*p* = 0.43	*p* = 0.043*		
*Developing vs. adult scaling*			Sig. different intercepts (*p* < 0.007**)					Sig. different intercepts (*p* < 0.019*)

Significant *p*-values (*p* < 0.05) are indicated with asterisks. When the overall ANCOVA did not reveal significant differences, no pairwise comparisons were conducted; when a significant interaction (i.e. slope difference) was found, no intercept differences were assessed. *M. eug. = Macropus eugenii, T. vulp. = Trichosurus vulpecula, M. dom. = Monodelphis domestica*.

*Hypothesis 3: Intraspecific growth patterns with age predictable by overall brain size, or growth reflecting important internal events*.

**Figure 5:**
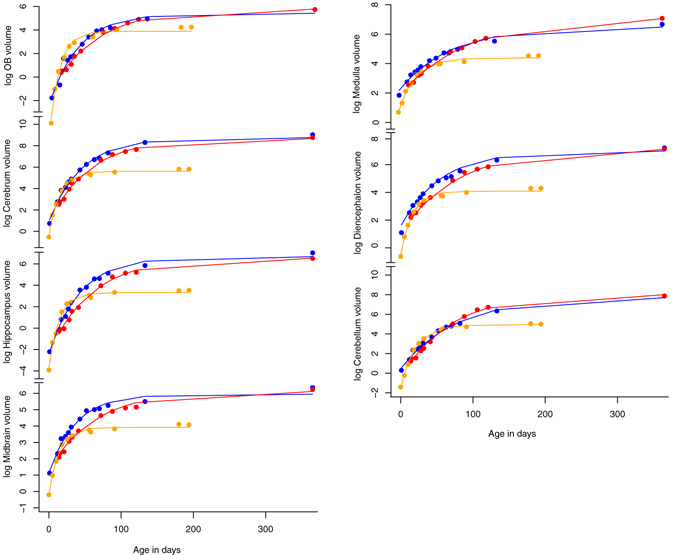
Plots of log partition volume growth (in mm^3^) against specimen age, with curves fitted as described in Materials and Methods.

Growth with age occurs in a regular pattern in all partitions within each species, with a steep initial growth phase followed by a plateau as the partitions reach adult size. This is typical for a hyperbolic von Bertalanffy growth curve^38^, which is described by the estimated time of growth commencement (t_0_), growth rate (k), and maximum estimated size (V_max_). Each species has a particular growth profile (Fig. 5, Table 3, for significance statistics, see Supplementary Table 3)., with growth rates (k) in particular mostly significantly different between species. Generally, partitions of the smallest-brained species, *M. domestica*, grow substantially faster (have high k) over a much shorter time frame than the other two species (indicated by nearly always a late t_0_ and early cessation of growth, see Fig. 5). V_max_ of *T. vulpecula* and *M. eugenii* are mostly not significantly different, meaning that adult partition sizes are similar enough to be within the error of the curve fit. However, these similar V_max_ are achieved through different growth patterns, with *M. eugenii* mostly having significantly higher growth rates (k) but shorter intervals of growth (see Fig. 5). A similar, intraspecifically regular but interspecifically varied pattern is also seen in non-log transformed plots of age *vs*. brain volume (Supplementary Fig. S3)

**Table 3:** Partition growth curve parameters of natural logarithm growth curves in the three species investigated.

	M. eugenii			T. vulpecula			M.domestica		
	V_max_	k	t_0_	V_max_	k	t_0_	V_max_	k	t_0_
Olf.bulb	5.42_Tv_	0.024	12.02_Tv_	5.80 _Me_	0.016	11.42_Me, Md_	3.89	0.072	9.16_Tv_
Cerebrum	8.77_Tv_	0.022	−4.98_Tv_	8.69 _Me_	0.018	−5.07_Me_	5.62	0.066	1.01
Hippoc,	6.68 _Tv_	0.023	13.34	6.56 _Me_	0.017	18.06	3.32	0.071	10.71
Dienceph.	7.00 _Tv_	0.018	−14.03 _Tv_	7.17 _Me_	0.013	−11.91 _Me_	4.09	0.062	1.95
Midbrain	5.95 _Tv_	0.027	−7.59 _Tv_	6.13 _Me_	0.016	−14.75 _Me_	3.92	0.069	0.72
Cerebellum	7.41 _Tv_	0.013_Tv_	−1.04	7.69 _Me_	0.014_Me_	7.18_Md_	4.59	0.048	7.07_Tv_
Medulla	6.51	0.014_Tv_	−28.14_Tv_	7.16	0.011_Me_	−24.91_Me_	4.39	0.043	−4.06

Vmax, maximum size estimate; k, growth rate; t0, day at which growth is estimated to have commenced. Species abbreviations in subscript denote parameters which are not significantly different compared to the other species (e.g. t_0_ of the olfactory bulb of *T.vulpecula* is not significantly different from that of *M. eugenii* and *M. domestica*).

Interestingly, estimates of the t_0_ parameter (the estimated time of growth commencement) vary substantially between species. This reflects a “forward”-and “backward”-shift of the entire growth curves along the x axis, indicative of heterochronic changes in overall growth timing. In particular, only the onset of medulla growth is estimated to have occurred before birth (negative t_0_) in *M. domestica*, whereas the growth of all but two partitions of *T. vulpecula* and *M. eugenii* are estimated to have begun antenatally. T_0_ estimates tend to be more similar in the more closely related *M. eugenii* and *T. vulpecula*. The most substantial onset heterochrony is between the cerebellum of *M. eugenii* and that of *T. vulpecula* and *M. domestica*. Despite its late neurogenetic schedule, the onset of growth in the cerebrum is not particularly late; for example, in all three species, it has an earlier t_0_ than the cerebellum. However, we caution that t_0_ estimates only appear to describe later-stage growth, as they do not describe antenatal growth patterns or growth at minute (~0.1 ml) partition volumes very well. In particular, the very late t_0_ of hippocampus growth (at 18 postnatal days in *T. vulpecula*, which is clearly incorrect) and the extremely early onset of medulla growth (at 28 pre-natal days in *M. eugenii*, nearly as long as the entire pregnancy) suggest that the earliest patterns of partition growth do not follow the same growth curve as the larger-scale postnatal growth patterns. The ages of eye opening and changes of milk composition are towards the end or after the rapid growth phase of all partitions in all species (Table 4).

**Table 4:** Timing of early developmental milestones (in postnatal days).

	Eyes open	Potassium drops/sodium increases	Carbohydrate levels drop	End of rapid growth phase
*M. eugenii*	168	~150	~220	~ 90
*T. vulpecula*	105	~110	~110	~100
*M. domestica*	44	~35	~55	~40

Eye opening data from Workman^25^ and references therein; milk composition data for *T. vulpecula from* Cowan^64^, for *M. domestica* from Green *et al*.^65^ and for *M. eugenii* from Sharp *et al*.^59^.

### Phylogenetic signal

The partition volume data are collated in Supplementary Data File S1. Most pgls models of partition scaling had high λ values (i.e. partition scaling patterns contain a large amount of phylogenetic signal), with only a few having very low λ. Where λ was estimated near 1 or near 0, W score comparisons (comparing the relative likelihood of estimated, λ = 1, and λ = 0 models out of one^39^) generally supported the result (green in Table 5). Using Grafen-transformed branch lengths yielded similar results but with higher levels ambiguity (for detailed statistics and the Grafen-transform analysis see Supplementary Tables S4,5). Phylogenetic signal varies between clades. Phylogenetic signal across all partitions was strongest within placental mammals (in datasets with and without olfactory bulb), followed by the placental subset of Primates. Marsupial partitions were most variable, as were eulipotyphlan and afrosoricidan partitions, possibly due to the very low sample sizes (n = 13/14) in these analyses.

**Table 5:** Pagel’s λ of phylogenetic generalized least squares analyses of partition volume against whole brain volume minus partition volume.

WBV-Partition	Marsupials	Placentals	Placentals without OB	Primates	Afrosoricidae	Eulipotyphla
*N*	28	75	104	45	14	13
Olf. bulbs	**1**	**0.93**	-	**0.84**	**1**	**1**
Cerebrum	**0.95**	**1**	**0.99**	**0.99**	0.4	**1**
Hippocampus	**1**	**0.92**	**0.99**	**0.96**	0.71	0
Diencephalon	0.63	**0.93**	**0.89**	0.86	**1**	0.63
Midbrain	0.59	**1**	**0.98**	**1**	0.63	0
Cerebellum	0.5	**0.97**	**0.92**	**0.86**	0	**1**
Medulla	0	**0.95**	**0.93**	0.43	**0.99**	0

Bold, high lambda and similar W scores as a model with λ = 1; regular font, ambiguous; underlined, low λ and similar W scores as a model with λ = 0 (see materials and methods for details).

## Discussion

The growth of brain partitions in the developing marsupials investigated here presents a diverse combination of patterns, with no support for the existence of interspecific regularities that could be attributed to a conserved neurogenetic schedule. Hypothesis 1 – predicting a conservative developmental scaling relationship between brain partitions of different species – was not supported, neither developmentally (only cerebrum and medulla are not significantly different in scaling between species) nor in comparisons between developing and evolving brain partitions. Hypothesis 2 – predicting regular scaling differences according to brain size, which might be attributable to neurogenetic processes - was also not supported because interspecific scaling slopes and intercepts follow no particular pattern that might be predictable from adult brain size. For example, the cerebellum of *M*. domestica, the species with the smallest brain, has a slope intermediate to the other two species; also, varying intercept differences suggest that growth commences from different starting points several partitions, which might also reflect the variable t_0_ estimates for commencement of partition growth curves (discussed under Hypothesis 3).

Hypothesis 3 – Interspecifically uniform growth with age, or growth according to important neurogenetic events – is also not supported. Each species has a distinctive partition growth profile, with the largest- and smallest-brained species in the sample (*M. eugenii* and *M. domestica*) generally having faster partition growth rates than the species with intermediate brain size (*T. vulpecula*). In addition, even with the caveats surrounding the relatively low resolution of our t_0_ (growth commencement) estimates, the early stages of growth appear to be extensively heterochronic between species, and do not seem to reflect neurogenetic scheduling, as the largest (and neurogenetically latest) partition is not particularly late in its growth onset. Without a more comprehensive understanding of how cell-level processes interact with the volumetric growth patterning of brain partitions, the source of this heterochrony is difficult to determine and needs further investigation, probably at the earliest patterning stages of brain development. However, our data suggest that paedo/peramorphosis in several brain growth parameters are probably responsible for the diversity of brain partition proportions among mammals^7^.

Within each species, the times corresponding to important cellular maturation processes within the brain (lactation regime change or eye opening) all occur towards the very end of the growth phase. There is thus no indication that some of the larger internal changes within the brain – neurogenesis-related or otherwise - are related to changes in growth patterns, such as growth spurts or lags.

The majority of scaling relationships between partition volume and whole brain volume carry very high phylogenetic signal, as identified by mostly very high Pagel’s λ values in the pgls analyses. This result is not unexpected, given that both brain development and partitions are known to show extensive mosaic evolution^15, 27, 40^. However, it further contradicts the suggestion that developmental constraints impact on the evolution of mammalian brain partitions. This also further highlights the extent of proportion plasticity in the mammalian brain, and confirms suggestions in the literature^7^ that mosaic evolution of relative brain partition sizes is widespread, substantial and biologically meaningful.

Aside from not supporting “late equals large” our results also highlight some statistical issues with the practice of correlating partitions against “rest of brain” or whole brain volume. One such issue is the impression of effect sizes: our ANCOVAs show substantial differences in slope or intercept between species in most developing brain partitions, but this is not obvious from the brain partition scaling plots (see Fig. 4). Overall, partition curves and slopes between the three species look very similar, an impression supported by high R^2^ values in the linear regressions of partition scaling including all three species (0.97 or above, with exception of the olfactory bulb). The impression might therefore arise that the statistical differences seen in the analyses reflect differences that are not biologically meaningful or due to negligible measurement error. However, the substantial differences between brain proportions of specimens at similar overall brain weights are visually obvious, even in the more closely related *M. eugenii* compared to *T. vulpecula* (compare in Fig. 3): For example, at similar brain volumes (around 2.3 ml), *M. eugenii* has half the cerebellar mass, 10% smaller medulla, but 45% larger midbrain, and 40% larger hippocampus, than *T. vulpecula*. This demonstrates how a visually tight regression fit, and high R^2^ values in regressions hide substantial absolute variation in our data. A similar issue was also raised in the literature with view to the suggestion that brain partition scaling across species is highly conserved, with some pointing out that the linear regression of partition volume against whole brain size hides considerable biological variation^15, 41, 42^. In the context of brain proportion evolution and development, retaining a reference to absolute differences therefore is an important component of investigation^14, 32^.

Regression of partitions against “rest of brain” of “whole brain” is also problematic because this method might use an allometrically confounded baseline. “Late equals large” was initially proposed based on scaling analysis of partition sizes against whole brain size, but after this approach was criticized for using part-whole correlations^15, 41, 42^, these scaling analyses are also often done through regressing brain parts against whole brain volume minus the partition of interest^17, 43^. If all brain partitions scaled predictably with brain size, this would not pose an issue. However, our results demonstrate substantial, species-specific scaling differences between most brain partitions and “rest of brain” (as well as whole brain volume). This is likely to introduce some noise; for example, we cannot exclude that the lack of significant interspecific differences between cerebrum and medulla scaling is an artifact of the combination of scaling patterns underlying the “rest of brain” measure. Similarly, the phylogenetic signal we found in our cross-species comparisons suggests scaling differences between mammalian clades, which might confound interspecific scaling comparisons. It is difficult to conceive of a better measure than “rest of brain” for testing brain partition scaling patterns, but the potential issues surrounding its use should be noted as a caveat in reports of brain vs. part-brain or whole-brain analyses^44^.

While our results do not preclude the existence of a mechanistic link between brain partition growth and neurogenesis, it would have to occur by neuronal development and cell partition volumes converging on a linked adult pattern, by a process we were not able to detect. However, it is interesting to note that the olfactory bulbs are least regular in terms of growth against brain volume as well as over time. This supports the suggestion that the olfactory bulb is the only part of the brain that is not, or only moderately, subject to the developmental constraints acting on the remainder of the brain^5, 8^. It is possible that this relates to a less integrated role of the olfactory bulb as receiving and transferring olfactory cues, as opposed to the more complex connections of other brain parts with each other. It is also consistent with studies showing that the neuronal density of the olfactory bulb differs between clades, and does not consistently scale with the remainder of the brain^30^. Whether this difference is due to a release from neurogenesis-based developmental scaling constraints, or due to a strong adaptive requirement for enhanced olfaction, remains to be established.

Despite little evidence for a neurogenesis-determined partition growth pattern, the regular brain partition growth with age suggests a robust developmental program at a time of complicated and extensive internal maturation processes and highly variable cell content^31, 36^. This could be explained with a neuromeric model of early^45^ brain partition patterning, which posits that the brain is divided through highly regional gene expression long before its partitions arise^45, 46^. This early patterning also appears to influence local neurogenesis^47, 48^. This would explain the regular growth patterns found in our data, which contrast with the highly variable cell and neuron densities found in the developing brain of *M. domestica* ^31^. It could even be speculated that the impression of neurogenesis-determined partition volume evolution reflects far earlier – and evolutionarily more flexible – partition patterning that also produces “matching” neurogenetic patterns. One could thus speculate that “late equals large”- like hypotheses and the neuromer-model could be integrated into a highly resolved, development-to-deep time view of mammalian (and vertebrate) brain partition evolution. Further testing can include more localized, sub-partition focused approach (e.g. as in Charvet *et al*.^28^), integrating the well-established negative scaling relationship of neuron density with adult brain size^28^, and the role of neuronal size^30^. Developmentally, matching comparative neuromer expression patterns with adult partition sizes is a promising yet labour-intensive way forward, as is the incorporation of glia and neuron origins and migration patterns^62, 63^ into hypotheses of brain partition evolution.

## Methods

### Specimens

Two relatively closely related Australian diprotodontian marsupials - the tammar wallaby *Macropus eugenii* (n = 13; 1–365 days old) and the brushtail possum *Trichosurus vulpecula* (n = 10; 14–365 days old)- and one less related South American opossum species (*Monodelphis domestica*, n = 12; 0–194 days old) were investigated (Supplementary Table S1). *M. domestica* specimens came from a breeding colony at the University of Melbourne. Four *M. eugenii* specimens came from a colony at the Canberra CSIRO Health and Biosecurity flagship; the remaining *M. eugenii* specimens and all specimens of *T. vulpecula* were from older collections at the University of Melbourne. All work was conducted with ethics approval: *M. eugenii* (ACT permit K1606, SBS/CSIRO/077/16), *T. vulpecula* (MAEC (Vic) License No. 06118), M. domestica (VIC permit 1111998). All collections were performed in accordance with the relevant guidelines and regulations. Specimens were fixed and stored in 10% formalin, which induces a small amount of distortion, with little further impacts on brain overall size and proportions over long storage periods^49^. Two duplicate *M. domestica* specimens (one adult and one juvenile) were used to assess the replicability of brain size between same-age specimens. Partition volumes for an adult *T. vulpecula* specimen from Pirlot (1981) were added to the data. All *M. domestica* and most *M. eugenii* specimens were of known age from breeding records. The remaining four *M. eugenii* specimens were aged using head length and age regressions by Marotte and James^50^. *T. vulpecula* specimens were aged using head length *vs*. age regressions by Lyne and Vernhagen^51^. A small error might thus be associated with the ages (around ±1 day for specimens younger than a month; around ±5 for older specimens based on visual inspection of the graphs in Lyne and Marotte^51^). We deemed this acceptable because this potential error is far smaller than the time spans considered and the intervals at which most specimens were sampled.

### IKI staining and CT scanning

We modified^34^ a protocol by Wong *et al*.^52^. Briefly, brain tissue is incubated with a gel precursor in liquid state, subsequently polymerized (“gelled”) and stained with an 1.75% iodine/potassium iodide solution^35^. Scanning was conducted at the Center for Advanced Imaging, University of Queensland (Inveon multimodel Siemens PET/CT scanner) or The University of New England (Vtomexs system GE Phoenix scanner). From these μCT scans, the brain was virtually dissected into its main components using the 3D software Mimics (Mimics Research v17.0, Materialise NV; Supplementary Fig. S1 for examples of scans and some dissections).

We determined the differential effect of staining on brain tissue distortion through preliminary comparison of gelled and non-gelled brain part volumes before and after iodine staining, as determined by μCT. For this, two formalin-preserved mouse brains cut into ten slices and separated into a control group (no hydrogel) and a treatment group (stabilised with hydrogel). These were CT-scanned prior to staining and after 4 days of staining in 1.75% IKI solution to assess volume differences. Shrinkage in untreated brain tissue stained with 1.75% IKI averaged 35.5%, with a maximum of 46%, with far less shrinkage (11.2% with a far lower range, a maximum of 16.1%). In both gelled and ungelled pieces, shrinkage was greatest in brain pieces containing olfactory bulb and cerebral cortex tissue, and least in pieces consisting of cerebellum tissue. This suggests some partition-specific differential tissue shrinkage, possibly related to cell content, exactly as observed in formalin-only based shrinkage^49^. While possibly the source of some small error due to the variable cell content of partitions during the earliest stages of development, differential shrinkage is therefore mostly expected to be consistent between partitions, with relatively little impact to be expected on volumetric comparisons.

### Virtual brain dissection

Nine non-overlapping brain partitions (Table 6) were selected for segmentation, and virtually separated using the segmentation tools of Mimics with the help of published brain atlases^36, 53^ (Supplementary Fig. 1). The partitions were left and right olfactory bulbs, left and right cerebral hemispheres, hippocampus, diencephalon, midbrain, cerebellum and medulla (Fig. 3). Volume data for the left and right olfactory bulbs and the two hemispheres of the cerebrum, which should be identical due to the symmetry of the brain, were compared using an ANOVA. This analysis revealed no significant differences between the two so they were combined, resulting in a total of seven partitions used for analysis. The spinal cord was cut from each virtual 3D specimen at its connection to the brain (a distinctive bend at the base of the brain) so that whole brain volumes were consistent across specimens. In the case of the two largest *M. eugenii* specimens (133 days and adult), the olfactory bulbs were lost in the dissection process. Estimates for these partitions were calculated from the proportion of olfactory bulb volume in the adult *M. eugenii* from Pirlot^54^. All segmentations were done by the same person (AC).

**Table 6:** The components included in each of the seven brain partitions used in the present study.

Brain partition	Components of partition
**Olfactory bulbs**	Includes anterior olfactory nuclei and glomeruli. Does not include nerve fascicles that surround the surface of the partition, or the ventricle inside the bulbs.
**Cerebrum**	Includes claustrum, cerebral white matter, paleocortex, septum, basal ganglia (including caudate, putamen and amygdala), internal capsule, anterior commissure, fasciculus aberrans, ectorhinal, entorhinal and perirhinal cortex. This relatively broad assembly of tissues was necessary because younger specimens lacked distinction between sub-regions of the cerebral cortex. Does not include lateral ventricles.
**Hippocampus**	Includes hippocampus proper, dentate gyrus and all subiculum region.
**Diencephalon**	Includes epithalamus, thalamus, hypothalamus, preoptic area, pretectum and optic chiasm. Does not include third ventricle.
**Midbrain**	Includes superior/inferior colliculus, periaqueductal grey and tegmentum.
**Cerebellum**	Includes all cerebellum proper, and middle cerebellar peduncle where present.
**Medulla (including pons)**	Segmented using distinctive differences in tissue density and borders apparent in 3D shape. Does not include cerebral peduncle or fourth ventricle.

The delineation of each partition was based on the marsupial brain atlases of Ashwell^36^.

### Published data from adult mammals

For comparison of developmental and adult brain scaling, brain volume and partition volume data were obtained from Pirlot^54^ for 27 marsupials (17 Australian and 9 American species). Although these datasets were based on histological section data, they are deemed compatible with our volume reconstruction because they were explicitly adjusted for shrinkage artifacts. To make Pirlot’s^54^ adult data comparable to the present study’s developmental data on the cerebral hemispheres we combined the subdivisions of the cerebral cortex, such as the paleocortex and basal ganglia, which were recorded separately by Pirlot^54^. For comparative purposes, we also included a brain partition dataset on placentals, which included eulipotyphlans, afrosoricidans, and primates^55^; this dataset was extended by a further 29 species through use of a dataset from Reep *et al*.^43^, which however did not have olfactory bulb volumes included.

### ANCOVA analyses

*Hypotheses 1,2: Conserved partition scaling between species mirrors evolutionary scaling, or non-uniform interspecific partition scaling, predictable by adult brain size*

All analyses were performed in R^56^. ANCOVAs of brain growth were used to test for Hypotheses 1 (that all growth patterns should be uniform) and 2 (that size-dependent significant scaling differences should occur) as outlined in Fig. 2. We computed ANOVA tables of regressions of log brain partition volume against log whole brain volume minus the partition volume (natural logarithm was used throughout). Subtracting partition volume from whole brain volume is preferred to avoid issues with part-whole correlations^5, 41, 44^, but because earlier work also regressed partition volume against whole brain volume^8^, this was also done. We used the *aov* function of R, with an interaction term of species to determine whether significant interactions (i.e. scaling slope differences) occurred between species:

log (Partition volume)~log(Whole brain volume- Partition volume)*Species;

Significant interaction terms were further investigated to determine which species differed using the “testInteraction” function of *phia* ^57^. This allowed us to determine whether any significant differences found were in a consistent sequence from smallest (*Monodelphis domestica*) to largest *(Macropus eugenii*) species. If no significant interactions were apparent, we dropped the interaction term to assess whether significant slope differences existed:

log (Partition volume)~log(Whole brain volume-Partition volume) + Species;

If significant slope differences were found, these were compared using the “TukeyHSD” function, again to look for consistent differences according to brain size.

*Hypothesis 3: Growth patterns with age predictable by overall brain size, or growth reflecting important internal events*.

To assess the growth of partitions over time, we fitted von Bertalanffy (hyperbolic) growth curves of natural-log transformed partition volumes against specimen age using the “nls“ function. Using *nlme* ^58^, we then assessed whether the curve parameters (t0 - the theoretical start time, K- the growth rate, and Vmax - the maximum size) of one species was significantly different from those of other species. In addition, two important early developmental milestones were given particular attention to assess whether they coincide with any partition growth patterns. These include eye opening, which is also the time at which neurogenesis is considered finished^25^ and also the first stage towards independence in marsupial pouch young, and change of milk from early lactational phase (high – potassium, high carbohydrate) to later lactational phase (high-sodium and high-protein/lipids). Early lactation is generally expected to cater for organogenesis and specifically brain growth^59^.

### Phylogenetic signal in evolutionary brain partition scaling

Phylogenetic trees for the species in the adult partition dataset were taken from Timetree.org ^60^, read into R, and matched to the dataset using *caper* ^61^. To test the prediction by “late equals large” that no or little phylogenetic signal should be apparent in the scaling pattern of adult marsupial brains, we used the “pgls” function of *caper* to determine Pagel’s λ^62^ for phylogenetic generalized least squares (pgls) analysis of log partition volume as a function of log brain volume. Pagel’s λ is close to one when a Brownian motion process along the phylogeny explains a large part of the scaling relationship between brain and partition volume, and close to zero if there is no relationship between the scaling relationship and the phylogeny^62^. High values of λ thus suggest strong phylogenetic signal. To determine the robustness of the λ values, we also computed alternative models in which λ was fixed at 1 and zero. For the three models thus obtained, we used the Akaike information criterion (AIC) values to compute W scores, which compare the likelihoods of several models relative to each other, with all likelihoods adding up to one^39^. Thus, if a model has strong phylogenetic signal, it is expected to have a high W score similar to the model with λ fixed at 1 (e.g. both estimated and λ = 1 model might have a score of 0.49) and a high W score compared to the model with λ = 0 (e.g. this model would have a score of 0.02). Models were deemed to show strong phylogenetic signal if the W score of the model with estimated λ, and the W score of the model with λ = 1, were similarly high (at least 0.75 in sum); they were deemed ambiguous when W scores were evenly distributed between all three models (e.g. each might have a value of 0.3); and they were deemed to have low or no phylogenetic signal if the W score of the model with estimated λ, and the W score of the model with λ = 0, were similarly high (at least 0.75 in sum). To account for the possibility that the tree branch configuration might provide spurious results, the pgls analysis was also conducted using Grafen’s branch length transform (using the “compute.brlen” command), which ultrametricises the tree based on theoretical expectations of cladogenesis^63^.

## Electronic supplementary material

Supplementary Materials

Supplementary Dataset 1
