# Supplementary material for: Testing hypotheses of developmental constraints on mammalian brain partition evolution, using marsupials
Source: Sci Rep. 2017 Jun 26;7:4241. doi: 10.1038/s41598-017-02726-9 (PMC5484667; doi:10.1038/s41598-017-02726-9)
Supplement: Supplementary file 1 — Supplementary Materials [file 41598_2017_2726_MOESM1_ESM.docx]

**Supplementary materials of “Testing hypotheses of developmental constraints on mammalian brain partition evolution, using marsupials”**

Alison Carlisle, Lynne Selwood, Lyn A. Hinds, Norman Saunders, Mark Habgood, Karine Mardon, Vera Weisbecker


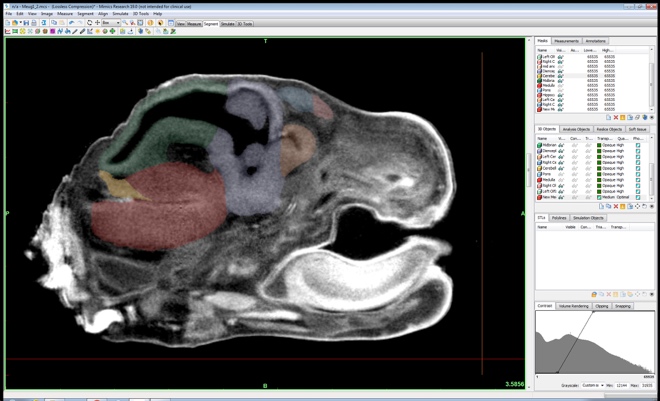

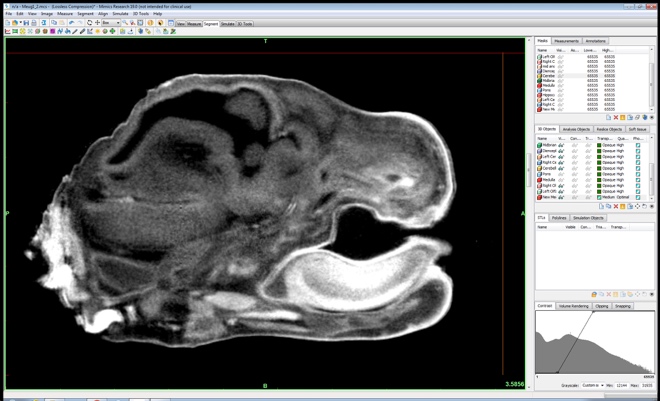

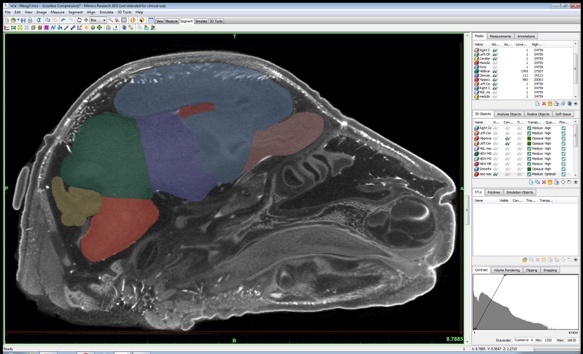

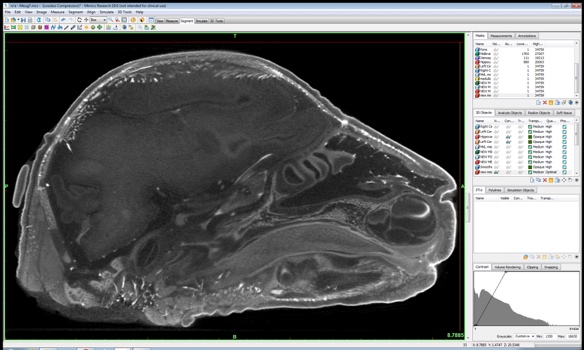

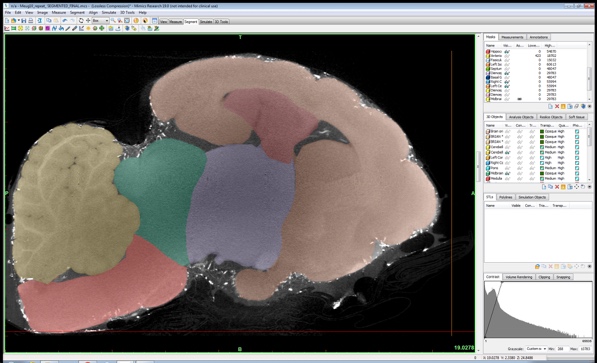

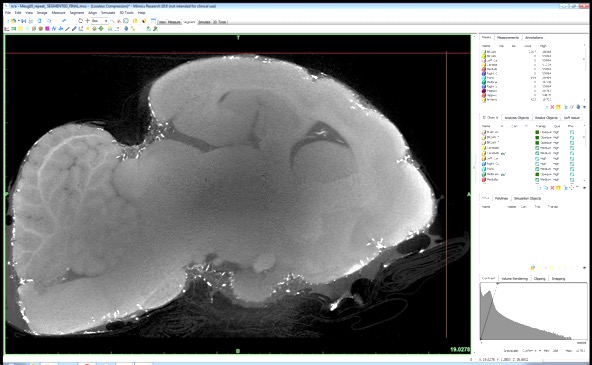

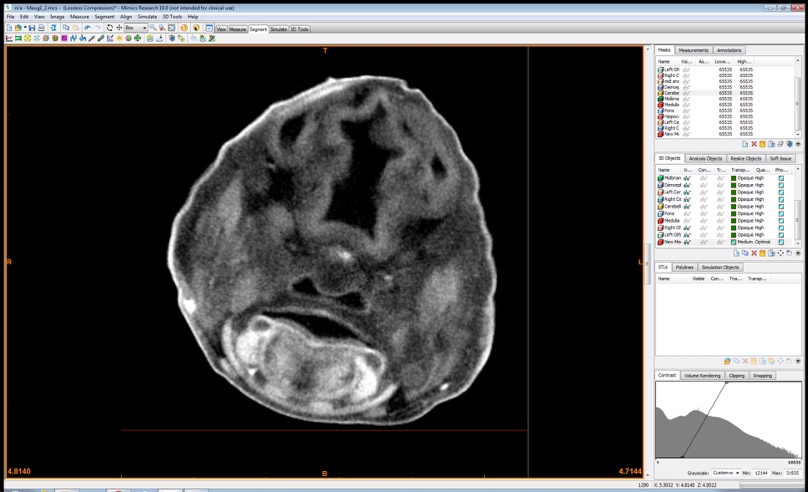

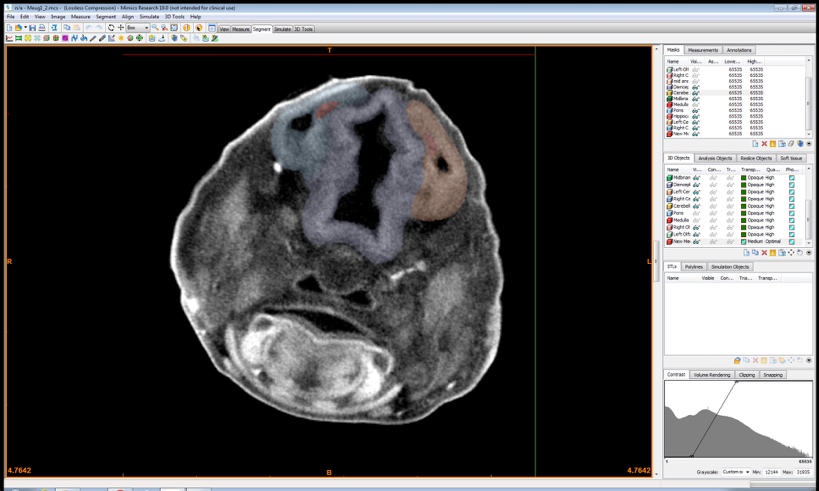

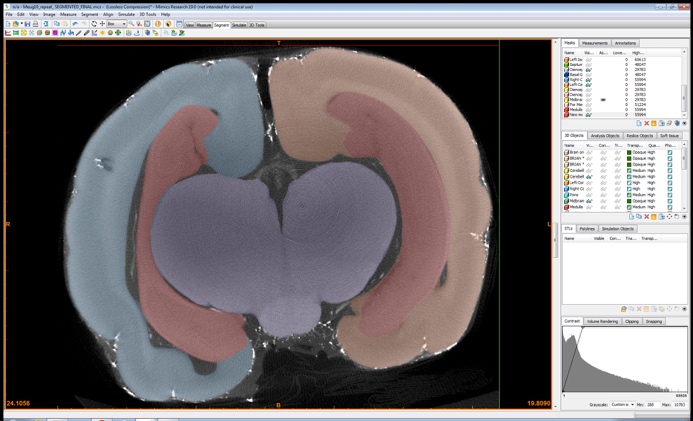

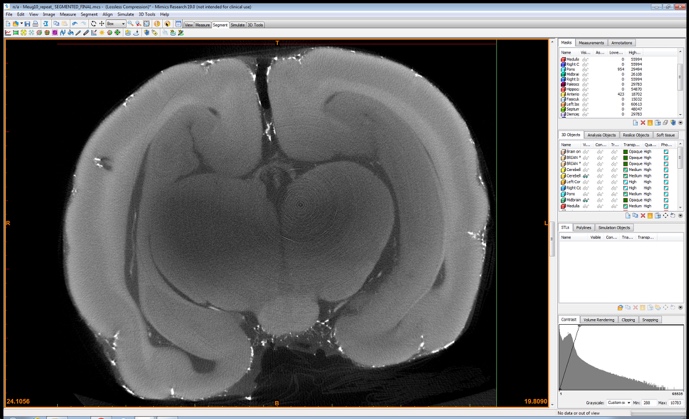

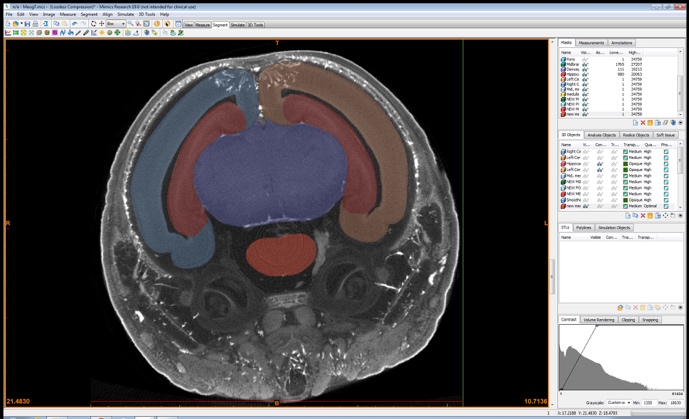

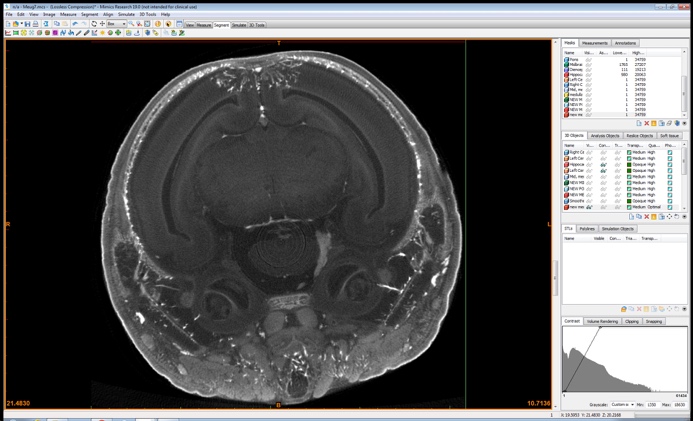


**1a**

**1b**

**2a**

**2b**

**3a**

**3b**

**1c**

**1d**

**2c**

2d

**3c**

**3d**

**Supplementary Figure S1:** 2D images of reconstructed uCT scan “slices” of the youngest (0 days; 1a-d; 60.24 pixels per mm), a mid-age (70 days; 2a-d; 35.84 pixels per mm), and the oldest and largest *M. eugenii* specimen (365 days; 3a-d; 35.84 pixels per mm), showing difference in scan resolution. Each specimen scan is represented by a coronal section (1a, 2a, 3a) and a sagittal section (1c, 2c, 3c). Coloured ‘masks’ of brain partitions are shown superimposed on the scans (1b, 2b, 3b, 1d, 2d, 3d).


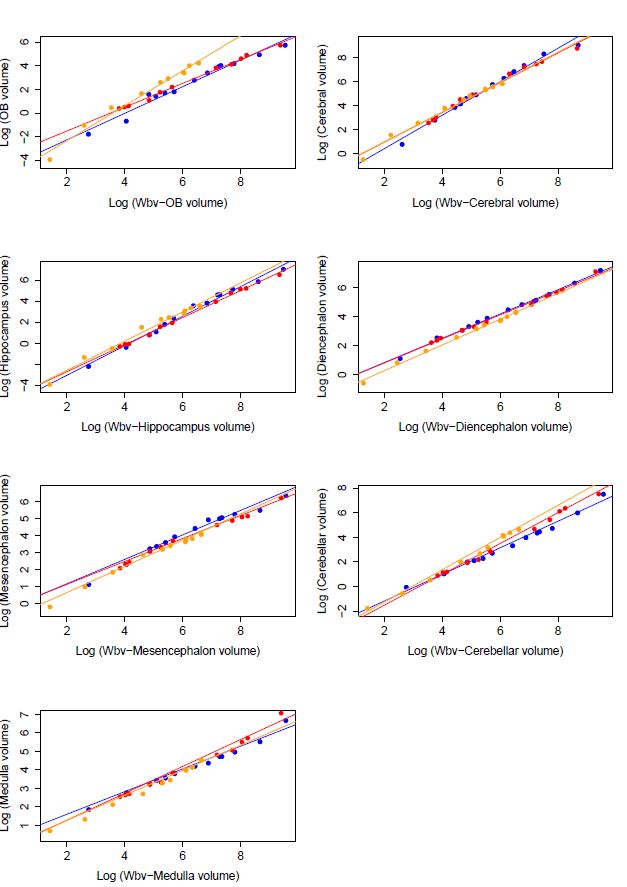


**Supplementary Figure S2:** Separate plots and linear fits of brain partition growth relative to whole brain volume (WBV) minus partition volume. Note that separate plots reveal more variation that the larger-scale joint plots.


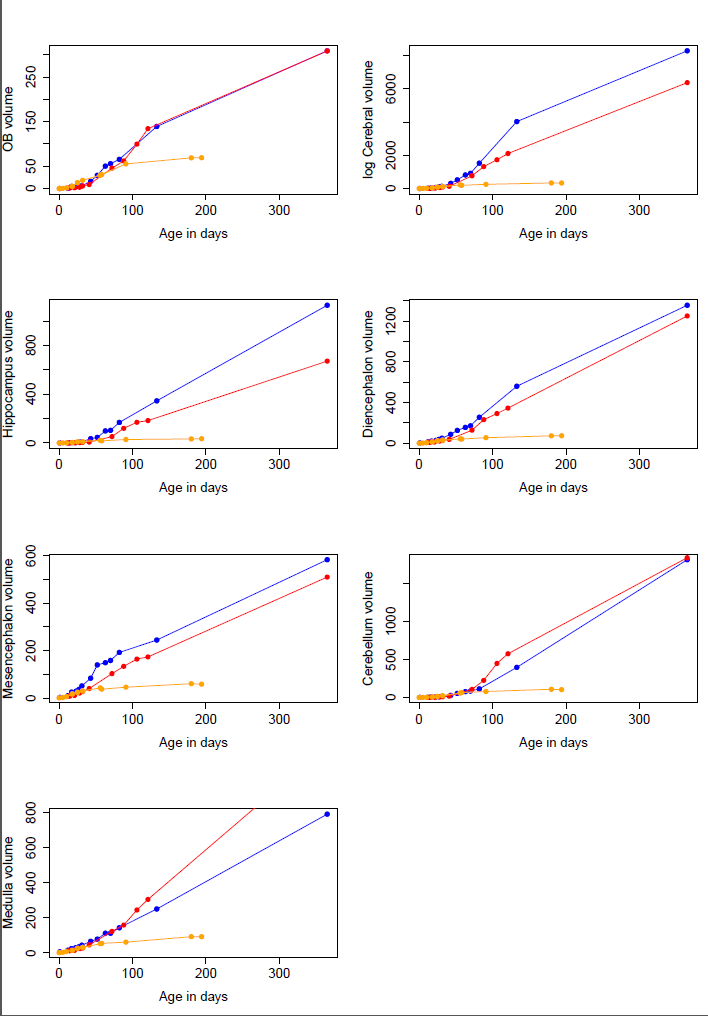


**Supplementary Figure S3:**  Growth plots of non-logarithm transformed brain partitions volumes (in mm^3^) vs. specimen age.

**Supplementary Table S1:** List of specimens with their ages, origins, and uCT scan resolution details. Specimens without asterisk were scanned by Karine Mardon at the Center for Advanced Imaging, University of Queensland, (Inveon multimodel Siemens PET/CT scanner). Specimens with an asterisk (*) were scanned by Stephen Wroe/Marie Attard at the University of New England (Vtomexs system GE Phoenix scanner).

| **Specimen ID** | **Age** | **Resolution (pixels per mm)** | **X, Y resolution at reconstruction** | **Z resolution at reconstruction** |
| --- | --- | --- | --- | --- |
| Meug 1 | 1 | 60.24096386 | 0.0166 | 0.0166 |
| Meug 2 | 17 | 60.24096386 | 0.0166 | 0.0166 |
| Meug 3 | 27 | 60.24096386 | 0.0166 | 0.033 |
| Meug 4 | 31 | 35.84229391 | 0.0279 | 0.0279 |
| Meug 5 | 43 | 35.84229391 | 0.0279 | 0.0279 |
| Meug 6 | 52 | 35.84229391 | 0.0279 | 0.056 |
| Meug 7 | 63 | 35.84229391 | 0.0279 | 0.056 |
| Meug 8 | 70 | 35.84229391 | 0.0279 | 0.056 |
| Meug 9 | 82 | 35.84229391 | 0.0279 | 0.056 |
| Meug 10 | 133 | 35.84229391 | 0.0279 | 0.055 |
| Meug 11 | adult | 35.84229391 | 0.0279 | 0.056 |
| Meug 1982 | 23 | 60.24096386 | 0.0166 | 0.033 |
| Meug 2839 | 12 | 60.24096386 | 0.0166 | 0.033 |
| Tvulp1 | 14 | 60.24096386 | 0.0166 | 0.0166 |
| Tvulp2 | 15 | 60.24096386 | 0.0166 | 0.0166 |
| Tvulp3* | 21 | 49.80079681 | 0.0201 | 0.0201 |
| Tvulp4 | 28 | 60.24096386 | 0.0166 | 0.033 |
| Tvulp5* | 32 | 45.35353077 | 0.0220 | 0.022 |
| Tvulp6 | 41 | 60.24096386 | 0.0166 | 0.033 |
| Tvulp7* | 72 | 27.7600422 | 0.0360 | 0.036 |
| Tvulp8* | 88 | 22.3478669 | 0.0447 | 0.045 |
| Tvulp9* | 106 | 16.66694445 | 0.0600 | 0.060 |
| Tvulp10* | 121 | 14.58470065 | 0.0686 | 0.069 |
| Mdom1* | 0 | 49.80079681 | 0.0201 | 0.0201 |
| Mdom2 | 5 | 60.24096386 | 0.0166 | 0.0166 |
| Mdom3 | 10 | 49.88277548 | 0.0200 | 0.0200 |
| Mdom4* | 18 | 60.24096386 | 0.0166 | 0.0166 |
| Mdom5 | 25 | 35.84229391 | 0.0279 | 0.0279 |
| Mdom6 | 32 | 35.84229391 | 0.0279 | 0.0279 |
| Mdom7 | 56 | 35.84229391 | 0.0279 | 0.0279 |
| Mdom8* | 58 | 27.7600422 | 0.0360 | 0.0360 |
| Mdom9* | 91 | 24.93454681 | 0.0401 | 0.0401 |
| Mdom10 | 194 | 36.10108303 | 0.0277 | 0.055 |
| Mdom11* | adult (2 years) | 20.03044628 | 0.0499 | 0.0499 |
|  |  |  |  |  |

**Supplementary Table S2:** Table of slope and intercept (Int.) *p*-values from ANCOVAs of partition volume and whole brain for comparisons between developing species. Significant *p*-values (*p*<0.05) are indicated with asterisks. Partitions that were not analysed are represented with a dash. *M. e.* = *Macropus eugenii, T. v. = Trichosurus vulpecula, M. d. = Monodelphis domestica,*  Int. = intercept.

| **Brain partition for Wbv~partiton comparisosn** | | | | | | | | | | | | | | | | |  |
| --- | --- | --- | --- | --- | --- | --- | --- | --- | --- | --- | --- | --- | --- | --- | --- | --- | --- |
|  |  | **ANCOVA** | | **Olfactory bulb** | **Cerebral cortex** | | **Hippocampus** | | **Diencephalon** | | **Midbrain** | | **Cerebellum** | | **Medulla** | | |
| **SPECIES** | **Slope** | ***All*** | *p*<0.0001 *** | | | *p*=0.163 | | *p*=0.149 | | *p*=0.096. | | *p*=0.111 | | *p*=0.001** | | *p*=0.0168* |  |
|  |  | ***M. e. – M. d.*** | *p*<0.0001 *** | | | **-** | | - | | - | | - | | *p*<0.0001*** | | *p*=0.46 |  |
|  |  | ***M. d. – T. v.*** | *p*<0.0001 *** | | | **-** | | - | | - | | - | | *p*=0.0001*** | | *p*=0.048 * |  |
|  |  | ***T. v. – M. e.*** | *p=*0.0364* | | | **-** | | - | | - | | - | | *p*=0.003 * | | *p*=0.154 |  |
|  | **Int.** | ***All*** | **-** | | | *p*=0.0508 | | *p*=0.001** | | *p*<0.0001*** | | *p*=0.00133** | | - | | *-* |  |
|  |  | ***M. e. – M. d.*** | **-** | | | **-** | | *p*=0.028* | | *p*<0.0001*** | | *p*=0.002** | | - | | - |  |
|  |  | ***M. d. – T. v.*** | **-** | | | **-** | | *p*=0.0018** | | *p*<0.0001*** | | *p*=0.391 | | - | | - |  |
|  |  | ***T. v. – M. e.*** | **-** | | | **-** | | *p*=0.403 | | *p*=0.414 | | *p*=0.049* | | - | | - |  |

|  | *M. eugenii* | | | | | *T. vulpecula* | | | | | | | *M.domestica* | | |
| --- | --- | --- | --- | --- | --- | --- | --- | --- | --- | --- | --- | --- | --- | --- | --- |
|  | **Vmax** | | **k** | **t0** | | **Vmax** | | **k** | | **t0** | | | **Vmax** | **k** | **t0** |
| Olf. bulb | 5.42_Tv_ | | 0.024 | 12.02_Tv_ | | 5.80 _Me_ | | 0.016 | | 11.42_Me, Md_ | | | 3.89 | 0.072 | 9.16_Tv_ |
| Cerebrum | 8.77_Tv_ | | 0.022 | -4.98_Tv_ | | 8.69 _Me_ | | 0.018 | | -5.07_Me_ | | | 5.62 | 0.066 | 1.01 |
| Hippoc. | 6.68_Tv_ | | 0.023 | 13.34 | | 6.56_Me_ | | 0.017 | | 18.06 | | | 3.32 | 0.071 | 10.71 |
| Dienceph. | 7.00_Tv_ | | 0.018 | -14.03_Tv_ | | 7.17_Me_ | | 0.013 | | -11.91_Me_ | | | 4.09 | 0.062 | 1.95 |
| Midbrain | 5.95_Tv_ | | 0.027 | -7.59_Tv_ | | 6.13_Me_ | | 0.016 | | -14.75_Me_ | | | 3.92 | 0.069 | 0.72 |
| Cerebellum | 7.41_Tv_ | | 0.013_Tv_ | -1.04 | | 7.69_Me_ | | 0.014_Me_ | | 7.18_Md_ | | | 4.59 | 0.048 | 7.07_Tv_ |
| Medulla | 6.51 | | 0.014_Tv_ | -28.14_Tv_ | | 7.16 | | 0.011_Me_ | | -24.91_Me_ | | | 4.39 | 0.043 | -4.06 |
| Comparison statistics | | | | | | | | | | | | | | | |
|  | |  | | | | | Vmax | | | | K | | | t_0_ | |
|  | | **Comparison between** | | | | | Diff. | | *P* | | Diff. | *P* | | Diff. | *p* |
| Olfactory Bulb | | **Meug - Mdom** | | | | | -1.533 | | **0.000** | | -0.048 | **0.000** | | 2.858 | **0.034** |
|  |  | **Mdom - Tvulp** | | | | | 1.912 | | **0.000** | | -0.056 | **0.000** | | 2.261 | 0.333 |
|  |  | **Meug - Tvulp** | | | | | 0.380 | | 0.355 | | -0.008 | 0.035 | | -0.596 | 0.811 |
| Cerebrum | | **Meug - Mdom** | | | | | 3.148 | | **0.000** | | -0.044 | **0.000** | | -5.991 | **0.000** |
|  |  | **Mdom - Tvulp** | | | | | 3.072 | | **0.000** | | -0.048 | **0.000** | | -6.082 | **0.004** |
|  |  | **Meug - Tvulp** | | | | | -0.076 | | 0.747 | | -0.004 | **0.049** | | -0.088 | 0.968 |
| Hippocampus | | **Meug - Mdom** | | | | | 3.353 | | **0.000** | | -0.049 | **0.000** | | 2.635 | **0.007** |
|  |  | **Mdom - Tvulp** | | | | | 3.233 | | **0.000** | | -0.054 | **0.000** | | 7.352 | **0.000** |
|  |  | **Meug - Tvulp** | | | | | -0.12 | | 0.702 | | -0.006 | **0.010** | | 4.716 | **0.001** |
| Diencephalon | | **Meug - Mdom** | | | | | 2.907 | | **0.000** | | -0.044 | **0.000** | | -13.85 | **0.000** |
|  |  | **Mdom - Tvulp** | | | | | 3.076 | | **0.000** | | -0.049 | **0.000** | | -15.98 | **0** |
|  |  | **Meug - Tvulp** | | | | | 0.168 | | 0.456 | | -0.005 | **0.007** | | -2.123 | 0.517 |
| Midbrain | | **Meug - Mdom** | | | | | 2.029 | | **0.000** | | -0.042 | **0.000** | | -8.300 | **0.000** |
|  |  | **Mdom - Tvulp** | | | | | 2.206 | | **0.000** | | -0.054 | **0.000** | | -15.465 | **0.000** |
|  |  | **Meug - Tvulp** | | | | | 0.177 | | 0.431 | | -0.012 | **0.001** | | -7.164 | 0.093 |
| Cerebellum | | **Meug - Mdom** | | | | | 2.823 | | **0.000** | | -0.034 | **0.000** | | -8.108 | **0.000** |
|  |  | **Mdom - Tvulp** | | | | | 3.102 | | **0.000** | | -0.033 | **0.000** | | 0.107 | 0.932 |
|  |  | **Meug - Tvulp** | | | | | 0.279 | | 0.292 | | 0.001 | 0.313 | | 8.217 | **0.000** |
| Medulla | | **Meug - Mdom** | | | | | 2.116 | | **0.000** | | -0.029 | **0.000** | | -24.07 | **0.000** |
|  |  | **Mdom - Tvulp** | | | | | 2.762 | | **0.000** | | -0.032 | **0.000** | | -20.85 | **0.000** |
|  |  | **Meug - Tvulp** | | |  | | 0.646 | | **0.007** | | -0.003 | 0.074 | | 3.225 | 0.502 |

**Supplementary Table S3:** **:** Partition growth curve parameters and comparison statistics of natural logarithm growth curves in the three species investigated. Species abbreviations in subscript denote parameters which are not significantly different compared to the other species (e.g. the three species have similar olfactory bulb growth patterns). Vmax, maximum size estimate; k, growth rate; t0, day at which growth is estimated to have commenced; Diff, difference in parameter values between the species compared; *p*, *p-*value of comparison.

**Supplementary Table S4:** Pagel’s λ of phylogenetic generalized least squares analyses of partition volume against whole brain volume minus partition volume (this is identical to Table 5 in the main manuscript, but replicated here for reference). W scores provide a measure of relative likelihoods of the three models compared; all three model likelihoods add up to one.

| WBV-Partition | Marsupials | | Placentals | Placentals without OB | | Primates | Afrosoricidae | | Eulipotyphla |  |
| --- | --- | --- | --- | --- | --- | --- | --- | --- | --- | --- |
| *N* | 28 | | 75 | 104 | | 45 | 14 | | 13 |  |
| Olf. bulbs | **1** | | **0.93** | - | | **0.84** | **1** | | **1** |  |
| Cerebrum | **0.95** | | **1** | **0.99** | | **0.99** | 0.4 | | **1** |  |
| Hippocampus | **1** | | **0.92** | **0.99** | | **0.96** | 0.71 | | 0 |  |
| Diencephalon | 0.63 | | **0.93** | **0.89** | | 0.86 | **1** | | 0.63 |  |
| Midbrain | 0.59 | | **1** | **0.98** | | **1** | 0.63 | | 0 |  |
| Cerebellum | 0.5 | | **0.97** | **0.92** | | **0.86** | 0 | | **1** |  |
| Medulla | 0 | | **0.95** | **0.93** | | 0.43 | **0.99** | | 0 |  |
| W score computations | | | | | | | | | |  |
| WBV-Partition | | W (λ estimated) | | | W (λ =1) | | | W (λ=0) | | |
| Marsupials | | | | | | | | | | |
| OB | | 0.50 | | | 0.50 | | | 0.00 | | |
| Cerebrum | | 0.57 | | | 0.43 | | | 0.00 | | |
| Hippocampus | | 0.49 | | | 0.49 | | | 0.02 | | |
| Diencephalon | | 0.61 | | | 0.11 | | | 0.28 | | |
| Midbrain | | 0.75 | | | 0.09 | | | 0.15 | | |
| Cerebellum | | 0.49 | | | 0.01 | | | 0.49 | | |
| Medulla | | 0.50 | | | 0.00 | | | 0.50 | | |
| Placentals | | | | | | | | | | |
| OB | | 0.92 | | | 0.08 | | | 0.00 | | |
| Cerebrum | | 0.50 | | | 0.50 | | | 0.00 | | |
| Hippocampus | | 0.67 | | | 0.33 | | | 0.00 | | |
| Diencephalon | | 0.88 | | | 0.12 | | | 0.00 | | |
| Midbrain | | 0.50 | | | 0.50 | | | 0.00 | | |
| Cerebellum | | 0.58 | | | 0.42 | | | 0.00 | | |
| Medulla | | 0.75 | | | 0.25 | | | 0.00 | | |
| Placentals without OB (Reep data) | | | | | | | | | | |
|  | | | | | | | | | | |
| OB | | - | | | - | | | - | | |
| Cerebrum | | 0.50 | | | 0.50 | | | 0.00 | | |
| Hippocampus | | 0.50 | | | 0.50 | | | 0.00 | | |
| Diencephalon | | 1.00 | | | 0.00 | | | 0.00 | | |
| Midbrain | | 0.65 | | | 0.35 | | | 0.00 | | |
| Cerebellum | | 0.98 | | | 0.02 | | | 0.00 | | |
| Medulla | | 0.97 | | | 0.03 | | | 0.00 | | |
| Primates | | | | | | | | | | |
|  | | | | | | | | | | |
| OB | | 0.86 | | | 0.14 | | | 0.00 | | |
| Cerebrum | | 0.50 | | | 0.50 | | | 0.00 | | |
| Hippocampus | | 0.51 | | | 0.49 | | | 0.00 | | |
| Diencephalon | | 0.76 | | | 0.23 | | | 0.00 | | |
| Midbrain | | 0.50 | | | 0.50 | | | 0.01 | | |
| Cerebellum | | 0.76 | | | 0.24 | | | 0.00 | | |
| Medulla | | 0.87 | | | 0.02 | | | 0.12 | | |
| Afrosoricidae | | | | | | | | | | |
| OB | | 0.46 | | | 0.46 | | | 0.07 | | |
| Cerebrum | | 0.47 | | | 0.14 | | | 0.39 | | |
| Hippocampus | | 0.51 | | | 0.29 | | | 0.19 | | |
| Diencephalon | | 0.47 | | | 0.47 | | | 0.06 | | |
| Midbrain | | 0.48 | | | 0.20 | | | 0.32 | | |
| Cerebellum | | 0.42 | | | 0.15 | | | 0.42 | | |
| Medulla | | 0.48 | | | 0.48 | | | 0.04 | | |
| Eulipotyphla | | | | | | | | | | |
| OB | | 0.50 | | | 0.50 | | | 0.00 | | |
| Cerebrum | | 0.48 | | | 0.48 | | | 0.05 | | |
| Hippocampus | | 0.49 | | | 0.02 | | | 0.49 | | |
| Diencephalon | | 0.53 | | | 0.14 | | | 0.33 | | |
| Midbrain | | 0.47 | | | 0.05 | | | 0.47 | | |
| Cerebellum | | 0.49 | | | 0.49 | | | 0.02 | | |
| Medulla | | 0.48 | | | 0.04 | | | 0.48 | | |

**Supplementary Table S5:** Pagel’s λ of phylogenetic generalized least squares analyses of partition volume against whole brain volume minus partition volume, using Grafen-transformed branch lenghts. W scores provide a measure of relative likelihoods of the three models compared; all three model likelihoods add up to one.

| WBV-Partition | Marsupials | | Placentals | Placentals without OB | | Primates | Afrosoricidae | | Eulipotyphla |  |  |
| --- | --- | --- | --- | --- | --- | --- | --- | --- | --- | --- | --- |
| *N* | 28 | | 75 | 104 | | 45 | 14 | | 13 |  |  |
| Olf. bulbs | 0.96 | | **0.96** | -- | | **0.84** | **0.82** | | **1** |  |  |
| Cerebrum | **0.98** | | **0.98** | **0.96** | | **0.89** | 0 | | 0.55 |  |  |
| Hippocampus | 0 | | 0.86 | **0.97** | | 0.61 | 0.64 | | 0 |  |  |
| Diencephalon | **1** | | **0.93** | **0.93** | | 0.79 | 0.80 | | 0.56 |  |  |
| Midbrain | 0.35 | | **0.97** | **0.97** | | 0.61 | 0.82 | | 0 |  |  |
| Cerebellum | 0 | | 0.89 | 0.88 | | 0.86 | 0 | | **1** |  |  |
| Medulla | 0 | | 0.88 | **0.92** | | 0.25 | **1** | | 0 |  |  |
| W score computations | | | | | | | | | |  |  |
| WBV-Partition | | W (λ estimated) | | | W (λ =1) | | | W (λ=0) | | |  |
| Marsupials | | | | | | | | | | |  |
| OB | | 0.67 | | | 0.33 | | | 0.00 | | |  |
| Cerebrum | | 0.55 | | | 0.45 | | | 0.00 | | |  |
| Hippocampus | | 0.49 | | | 0.01 | | | 0.49 | | |  |
| Diencephalon | | 0.47 | | | 0.47 | | | 0.06 | | |  |
| Midbrain | | 0.67 | | | 0.02 | | | 0.31 | | |  |
| Cerebellum | | 0.57 | | | 0.43 | | | 0.00 | | |  |
| Medulla | | 0.50 | | | 0.01 | | | 0.50 | | |  |
| Placentals | | | | | | | | | | |  |
| OB | | 1.00 | | | 0.00 | | | 0.00 | | |  |
| Cerebrum | | 0.79 | | | 0.21 | | | 0.00 | | |  |
| Hippocampus | | 1.00 | | | 0.00 | | | 0.00 | | |  |
| Diencephalon | | 1.00 | | | 0.00 | | | 0.00 | | |  |
| Midbrain | | 0.99 | | | 0.01 | | | 0.00 | | |  |
| Cerebellum | | 1.00 | | | 0.00 | | | 0.00 | | |  |
| Medulla | | 1.00 | | | 0.00 | | | 0.00 | | |  |
| Placentals without OB (Reep data) | | | | | | | | | | |  |
| Cerebrum | | 1.00 | | | 0.00 | | | 0.00 | | |  |
| Hippocampus | | 1.00 | | | 0.00 | | | 0.00 | | |  |
| Diencephalon | | 0.00 | | | 1.00 | | | 0.00 | | |  |
| Midbrain | | 1.00 | | | 0.00 | | | 0.00 | | |  |
| Cerebellum | | 1.00 | | | 0.00 | | | 0.00 | | |  |
| Medulla | | 1.00 | | | 0.00 | | | 0.00 | | |  |
| Primates | | | | | | | | | | |  |
| OB | | 1.00 | | | 0.00 | | | 0.00 | | |  |
| Cerebrum | | 0.92 | | | 0.08 | | | 0.00 | | |  |
| Hippocampus | | 1.00 | | | 0.00 | | | 0.00 | | |  |
| Diencephalon | | 0.98 | | | 0.00 | | | 0.02 | | |  |
| Midbrain | | 0.34 | | | 0.00 | | | 0.66 | | |  |
| Cerebellum | | 1.00 | | | 0.00 | | | 0.00 | | |  |
| Medulla | | 0.78 | | | 0.00 | | | 0.22 | | |  |
|  | |  | | |  | | |  | | |  |
|  | |  | | |  | | |  | | |  |
|  | |  | | |  | | |  | | |  |
| Afrosoricidae | | | | | | | | | | |  |
| OB | | 0.59 | | | 0.30 | | | 0.11 | | |  |
| Cerebrum | | 0.47 | | | 0.06 | | | 0.47 | | |  |
| Hippocampus | | 0.73 | | | 0.08 | | | 0.19 | | |  |
| Diencephalon | | 1.00 | | | 0.00 | | | 0.00 | | |  |
| Midbrain | | 0.58 | | | 0.27 | | | 0.14 | | |  |
| Cerebellum | | 0.48 | | | 0.04 | | | 0.48 | | |  |
| Medulla | | 0.48 | | | 0.48 | | | 0.04 | | |  |
| Eulipotyphla | | | | | | | | | | | Eulipotyphla |
| OB | | 1.00 | | | 0.00 | | | 0.00 | | |  |
| Cerebrum | | 0.92 | | | 0.08 | | | 0.00 | | |  |
| Hippocampus | | 1.00 | | | 0.00 | | | 0.00 | | |  |
| Diencephalon | | 0.98 | | | 0.00 | | | 0.02 | | |  |
| Midbrain | | 0.34 | | | 0.00 | | | 0.66 | | |  |
| Cerebellum | | 1.00 | | | 0.00 | | | 0.00 | | |  |
| Medulla | | 0.78 | | | 0.00 | | | 0.22 | | |  |
